# Benefits for emotional regulation of contact with nature: a systematic review

**DOI:** 10.3389/fpsyg.2024.1402885

**Published:** 2024-07-19

**Authors:** María Luisa Ríos-Rodríguez, Christian Rosales, Bernardo Hernández, Maryurena Lorenzo

**Affiliations:** ^1^Department of Social Psychology, Social Work, Social Services and Social Anthropology, University of Málaga, Málaga, Spain; ^2^Department of Cognitive, Social and Organizational Psychology, University of La Laguna, San Cristóbal de La Laguna, Spain; ^3^Department of Communication Sciences and Social Work, Faculty of Social Sciences and Communication, Universidad of La Laguna, San Cristóbal de La Laguna, Spain

**Keywords:** connectedness to nature, nature contact, emotional regulation, systematic review, emotional self-regulation, emotional management

## Abstract

**Introduction:**

Exposure to natural environments, such as parks, forests, and green areas, is often linked to a decrease in stress, anxiety and depression, while contributing to improved emotional wellbeing. These observations are supported by well-established theories, such as the Stress Reduction Theory and Attention Restoration Theory, which highlight the psychological benefits of interacting with nature. However, the relationship between exposure to nature and emotions, and in particular, with emotional regulation, is an evolving aspect of research with no clear conclusions. Emotional regulation can be deliberate in nature, where individuals voluntarily participate in modifying various aspects of their emotions, such as their type, intensity, quality or duration. Alternatively, it may be automatic, originating from sensory perception and acting without full awareness, but significantly influencing emotional experiences. In this context, the environmental self-regulation hypothesis, suggests that people consciously or unconsciously use their physical environment to regulate their emotions.

**Method:**

To analyze the evidence of the relationship between contact with nature and emotional regulation, we conducted this review. Using the PRISMA statement as a reference, we conducted keyword searches in five databases in the period between 2013 and 2023. The databases selected were Scopus, Web of Science (WoS), PubMed, PsycINFO and ScienceDirect.

**Results:**

In addition, a manual search was carried out of journals in the research field. Initially, from which gray literature, reviews and duplicates were removed in a first step. The resulting articles were then filtered using their titles and abstracts. Subsequently, the abstracts of the 25 selected articles were reviewed and discussed by researchers to reach a final decision based on consensus about the adequacy of each paper. Finally, nine articles were included in the systematic review.

**Discussion:**

In general terms, this review suggests that research on the relationship between contact with nature and emotional regulation provides valuable insights into how natural environments can contribute to the emotional wellbeing and physical and mental health of the population.

## 1 Introduction

Emotion regulation involves attempts to influence one's own or others' emotions. In recent decades, emotional regulation has gained notable prominence in various subdisciplines of psychology (McRae and Gross, 2020). The current relevance of emotional regulation lies in its significant impact on physical and mental health, as well as psychological wellbeing. This phenomenon also influences the quality of social relationships, the learning process and academic performance (Gross and John, 2003; Graziano et al., 2007; Twohig-Bennett and Jones, 2018).

There are significant mental health needs worldwide. However, existing responses to these needs are few and inadequate. According to recent data, one in eight people in the world suffers from a mental disorder, with mood and anxiety disorders being the most prevalent (WHO, 2022). The report on mental health in the world highlights that to achieve the goals proposed in the WHO's Comprehensive Mental Health Action Plan 2013–2030 and the Sustainable Development Goals, it is necessary to transform the environment, as it has the capacity to influence on our mental health (Tomasi et al., 2020; WHO, 2021).

Research on emotional regulation has mainly focused on two specific strategies. First, cognitive reappraisal, which involves cognitive changes that reinterpret emotion-generating situations, thus altering their emotional impact. Second, expressive suppression, which consists of inhibiting emotional expressions (Gross, 2015). Gross and Thompson's (2007) emotional regulation model is a theoretical framework that identifies five emotion regulation strategies that occur during different moments of an emotional experience: (1) situation selection involves choosing environments that are likely to generate positive emotions and avoiding those that may cause negative ones; (2) situation modification consists of altering the situation to change its emotional impact; (3) attention deployment refers to directing attention toward or away from certain stimuli to influence the emotions that are experienced; (4) cognitive change involves reinterpreting a situation to alter its emotional meaning; and (5) response modulation covers regulating the expression of emotions to conform to social demands. This model provides a framework to understand how people regulate their emotions to adapt to social demands, thereby influencing their emotional wellbeing and social adaptation.

Research relating to emotions and nature has been supported by two theories: Attention Restoration Theory (ART, Kaplan and Kaplan, 1989; Kaplan, 1995) and the psychophysiological Stress Reduction Theory (SRT, Ulrich, 1993). ART posits that exposure to natural environments can restore attentional capacity, reducing mental fatigue and improving concentration. The theory identifies four key components for this restoration: being away, extent, fascination, and compatibility. Additionally, it suggests that nature provides a type of “soft” fascination that allows cognitive recovery without conscious effort, which is crucial for mental restoration. Interaction with nature is considered essential for psychological wellbeing and mental health. In contrast, SRT posits that humans have a genetic predisposition to prefer certain natural environments, such as green and open landscapes, due to evolution. This innate preference translates into stress reduction when people are exposed to these natural settings. According to SRT, exposure to nature can decrease physiological and psychological arousal, including reductions in blood pressure, heart rate, and stress hormone levels. This theory suggests that natural environments act as an antidote to modern stress, providing a calming effect that enhances overall wellbeing and mental health. These theories provide useful conceptual frameworks to understand how nature can have a positive impact on our emotions and psychological processes. In line with the above, the biophilia hypothesis suggests that the innate connection between humans and nature could be encoded in human genes. This biological affinity developed over evolution, as our ancestors relied on natural environments for survival (Kellert and Wilson, 1993). Taken together, these theories provide a solid foundation for investigating how nature can be used as an effective tool to regulate human emotions and improve emotional wellbeing. And specifically, the biophilia hypothesis implies that this innate connection with nature remains present in modern humans, influencing our preferences and behaviors. This may explain why many people find peace and restoration in natural environments and why exposure to nature has positive effects on our mental and emotional health.

Since the 19th century, it has been recognized that green spaces have benefits for the health of the population by providing opportunities for physical activity and the construction or maintenance of social relationships, among other aspects (Twohig-Bennett and Jones, 2018). Despite this awareness, the importance of nature in emotional regulation has often been underestimated. Several studies have highlighted the emotional benefits of contact with nature, although doubts remain about the underlying mechanisms (Gu et al., 2023). Thus, according to Capaldi et al. (2014), there is a relationship between being connected with nature and feeling happy.

Recent research highlights that for individuals experiencing anxiety or depression, spending time in natural or outdoor environments ranks as one of the three most supportive strategies to improve their wellbeing, along with adopting healthy behaviors and communicating with friends or family members (WHO, 2021). Thus, facilitating people's access to nature can contribute to their wellbeing and psychological health (Johnsen and Rydstedt, 2013). In addition, there are studies that emphasize the human need for affiliation and connection to the natural world (Mayer and Frantz, 2004). These studies indicate that people have an implicit connection with nature and its cognitive, affective and conative components (Schultz, 2002), as well as the mediating role of emotional regulation between nature and wellbeing (Richardson and McEwan, 2018).

Historically, sensations and emotions have been closely related. However, there is still much to explore in terms of how sensory experiences can promote emotional regulation. The role that the senses can play in managing emotions is often overlooked, even though they allow us to quickly detect information about the environment. Along these lines, it is essential to recognize that sensations can be used as a tool to regulate emotions, not only passively but voluntarily, activating our senses to strategically regulate our emotions (Rodriguez and Kross, 2023). In other words, emotion regulation can be automatic, originating in sensory perception and acting without full consciousness, but significantly influencing the emotional experience. It can also be deliberate in nature, in which individuals voluntarily participate to modify various aspects of their emotions, such as their type, intensity, quality or duration. In this sense, the environmental self-regulation hypothesis (Korpela, 1992; Korpela et al., 2020), suggests that individuals interact with their physical environment in ways that go beyond mere functionality or aesthetics. According to this theory, the physical environment can serve as an active tool to regulate emotions. This implies that individuals can consciously or unconsciously choose environments that help them improve their emotional state or manage their emotions more effectively. In other words, the environmental self-regulation hypothesis suggests that individuals are not only influenced by their environment, but also have the ability to influence their own emotional state through interaction with their physical surroundings. In addition, current research highlights the importance of distinguishing between environmental characteristics (such as the amount of vegetation, exposure to sunlight, etc.) and individual factors that promote a positive connection between people and their environment (Spano et al., 2023; Rosales et al., 2024).

Considering the above, the following research question arises: what is the relationship between contact with nature and emotional regulation? To address this issue, the following systematic review is presented to examine the evidence for the relationship between contact with nature and emotional regulation, understanding contact with nature as both direct and indirect interaction with natural environments and elements, in both urban and rural contexts. Regarding emotional regulation, it is important to understand its role as a dependent variable and comprehend how contact with nature can affect individuals' ability to regulate their emotions. In addition, there is the mediating role between contact with nature and other psychological or behavioral variables. In addressing this question, we seek to consolidate and critically evaluate available research and identify gaps in current knowledge. This work explores the implications of the findings for their repercussions on theories and research, promoting a deeper understanding of the connection between the natural environment and emotional regulation. Likewise, we aim to offer a comprehensive perspective that contributes to the development of nature-based therapeutic interventions.

## 2 Materials and methods

### 2.1 Databases and search strategy

A comprehensive search was conducted of several databases, including WOS, Scopus, PsycInfo, PubMed, and ScienceDirect, on 23 October, 2023. Search strategies were designed to include relevant terms related to nature and emotion regulation. Specifically, the following search string was employed in each database: “connectedness to nature” OR “nature contact” OR “exposure to nature” OR “urban nature” OR “proximity to nature” OR “nature connection” OR “nature connectedness” OR “park” OR “garden” OR “natural environment” OR “greenspace” OR “public space” AND “emotional regulation” OR “managing emotion” OR “emotional management” OR “emotional self-regulation” OR “emotion regulation”.

### 2.2 Data extraction and assessment process

**Figure 1:**
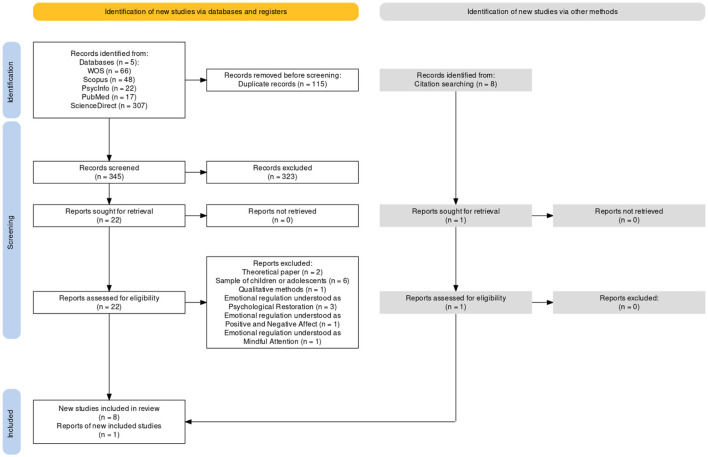
Flowchart.

This systematic review adhered to the PRISMA (Preferred Reporting Items for Systematic Reviews and Meta-Analyses) guidelines (Page et al., 2021). The initial search yielded 460 records, of which 115 duplicates were removed. In addition, another eight additional articles were identified through text references. Subsequently, researchers independently and simultaneously reviewed the titles and the abstracts of the 345 records. To facilitate collaboration and analysis, the results were compiled into an Excel file. Following this, a discussion among the four researchers was initiated to assess the relevance of the selected titles. Works in which there was either 100% or 75% agreement among the researchers were retained, while those with less consensus were subject to debate until a unanimous decision was reached. From this screening process, 323 records were discarded. Next, the 22 selected articles were reviewed. Once again, researchers discussed these articles and ones selected through other methods to arrive at a final decision based on consensus. Ultimately, nine articles have been included in this systematic review. Figure 1 presents the flow diagram, which has been designed using the app for PRISMA 2020-compliant flow diagrams (Haddaway et al., 2022).

### 2.3 Inclusion and exclusion criteria

The articles selected for review had to meet the following inclusion criteria:

- Articles had to be empirical works published in English that had undergone a peer-review process and had full-text access.
- Their publication date had to be after 2013. Limiting the review to the last 10 years can help focus on the most recent and relevant trends, which is particularly useful in rapidly evolving fields. In addition, this criterion was included because, from a practical standpoint, the authors decided to cover a more manageable and recent period to ensure a thorough review within the available limits.
- The selected articles must address “contact with nature” as the interaction, either directly or indirectly (e.g., images, photographs, videos, virtual reality, etc.) with natural environments and elements, both in urban and rural contexts. By diversifying the forms of contact with nature, our review aims to capture a broader spectrum of human-nature interactions and their potential effects on emotional regulation.
- This review includes studies that examine the role of emotional regulation as a mediating variable and outcome. Including studies that address both roles of emotional regulation enriches our review by providing a comprehensive view of how it is influenced and, in turn, influences other aspects of human behavior.
- The studies had to involve adult participants. We have opted for works with adult populations because emotional regulation, as the ability to modulate emotional experiences in a way that promotes emotional and intellectual growth, follows a course of development that extends and consolidates in adulthood (Gross, 2015).
- The studies had to use correlational, cross-sectional, experimental, and quasi-experimental designs. This decision is because these design types cover a broad spectrum of robust methodologies for evaluating relationships between variables and effects in controlled and natural contexts.

The reasons for excluding an article were as follows:

- Full text was not available.
- Publication in a language other than English and/or before 2013.
- The study sample consisted of children or adolescents. Children and adolescents are in a phase of continuous development, which means that their capacities for emotional regulation, cognitive processing, and psychological maturity significantly differ from those of adults (Theurel and Gentaz, 2018; Sanchis-Sanchis et al., 2020). This difference can influence how they interpret and manage their emotions. Moreover, excluding children and adolescents allows the review to maintain greater homogeneity in the participant profile, which facilitates comparison and synthesis of data.
- Qualitative methods were used. Qualitative studies may not be appropriate because their results are not quantifiable in the same way as the results of quantitative studies. Furthermore, they tend to explore more subjective and contextual aspects, which could introduce variables that are difficult to compare directly with quantitative results.
- It was a theoretical or review paper.

### 2.4 Quality and risk bias of selected studies

To assess the methodological quality of the studies included in this systematic review, each researcher evaluated the risk of bias using an Excel spreadsheet. The research group used the Joanna Briggs Institute (JBI) Critical Appraisal Checklist for Analytical Cross-Sectional Studies (Moola et al., 2020). Overall, the results of the assessments for the nine studies included in this systematic review were positive. Table 1 presents the percentage of agreement among the four researchers for each of the studies across different items of the JBI Checklist for Analytical Cross-Sectional Studies (Moola et al., 2020). The average degree of agreement among researchers regarding the analysis of bias risks for the nine included studies is above 70%.

**Table 1:** The percentage of agreement among the researchers in the analysis of bias risks.

**References**	**1**	**2**	**3**	**4**	**5**	**6**	**7**	**8**	**Average degree of agreement**
**(1)** Johnsen (2013)	50	100	100	75	50	50	100	100	78.13 (include)
**(2)** Johnsen and Rydstedt (2013)	50	75	75	75	50	50	100	100	71.89 (include)
**(3)** Bakir-Demir et al. (2021)	100	100	NA	100	100	100	100	100	100 (Include)
**(4)** Fido et al. (2020)	50	100	100	50	75	75	100	100	81.25 (include)
**(5)** Korpela et al. (2020)	75	75	75	50	100	100	100	100	84.38 (include)
**(6)** Richardson and McEwan (2018)	75	100	50	50	50	50	100	100	71.89 (include)
**(7)** Sallay et al. (2023)	100	50	NA	NA	NA	NA	100	100	87.5 (include)
**(8)** Theodorou et al. (2023)	100	100	100	100	100	100	100	100	100 (include)
**(9)** Zhang et al. (2022)	75	100	75	100	100	100	100	100	93.75 (include)

1: Were the criteria for inclusion in the sample clearly defined?; 2: Were the study subjects and the setting described in detail?; 3: Was the exposure measured in a valid and reliable way?; 4: Were objective, standard criteria used for measurement of the condition?; 5: Were confounding factors identified?; 6: Were strategies to deal with confounding factors stated?; 7: Were the outcomes measured in a valid and reliable way?; 8: Was appropriate statistical analysis used?

NA, Not Applicable.

## 3 Results

### 3.1 Characteristics of the examined studies

The characteristics of the included studies, data analysis and main results are presented in the Supplementary material. To refer to the articles, the numbering assigned in Figure 1 to each of the selected articles was used. The studies had an average sample size of 339.27 participants, with variations ranging from 35 (2) to 977 (9). Nine of the works analyzed used a survey data collection design, either for descriptive purposes or to establish causal relationships among the evaluated variables. Additionally, two studies adopted an experimental design, using randomization in group formation. Johnsen and Rydstedt (2013) employed an experimental design that differentiated three groups (experimental, control, and experimental-softer version). This research lasted for 2 weeks and used printed images as the independent variable for different stimuli in each group. The stimuli included natural environments, balloons, and natural environments with looser instructions, respectively. Measurements were taken at three different time points. In the study by Theodorou et al. (2023), four conditions were established, where each participant was presented with a virtual reality experience in various environments (urban, park, lake, and arctic). Each participant was assigned to one of the experimental conditions, with measurements taken before and after exposure. This study also emphasized the control of confounding variables.

Regarding the countries of origin for the nine selected studies, two of them were carried out in Norway (1, 2), another two in the United Kingdom (4, 6), and in two studies, data were collected in both Finland and Hungary (5, 7). The remaining studies were conducted in Turkey (3), Italy (8), and Singapore (9). These nations exhibit notable disparities in cultural dimensions such as the degree of value assigned to individualism, long-term orientation, masculinity, and uncertainty avoidance, according to the dimensions proposed by Hofstede (2001).

In the statistical treatment of the data, it is observed that several studies included exploratory and confirmatory factor analysis to validate some of the measures employed (1, 5, 7). Additionally, three studies tested causal models using structural equation modeling (1, 5, 9) or assessing mediation or moderation relationships between variables (3, 4, 6, 8). Two studies used correlational tests, such as mean comparison, regression analysis, or latent profile analysis (2, 7).

Finally, the studies approaches to emotional regulation vary. Some studies propose that emotional regulation acts as a mediating variable, for example, between personality traits and nature contact in relation to attention restoration (Johnsen, 2013). It is also analyzed as a mediator between nature connection and stress (Bakir-Demir et al., 2021) or as a mediator between motives for visiting natural spaces and that natural contact, in relation to effects on physical and mental health (Korpela et al., 2020; Zhang et al., 2022; Sallay et al., 2023; Theodorou et al., 2023). Conversely, other studies position emotional regulation as a dependent variable that is influenced by the environment or the use of the environment (Johnsen and Rydstedt, 2013; Richardson and McEwan, 2018; Fido et al., 2020).

### 3.2 Descriptive characteristics of the participants

A total of 3,732 individuals participated in the nine studies selected for this review. The age range varied from 16 to 85 years old, with a mean age of 28.6. However, not all studies reported the same descriptive data for joint evaluation (Table 2).

**Table 2:** Descriptive data of the examined samples.

	**Sample size**	**Sex**	**Age**		
	***N***	**Women**	**Range**	***M***	***SD***
**(1)** Johnsen (2013)	142	52.1%	16–79 years old	Median 40–49	They do not contribute
**(2)** Johnsen and Rydstedt (2013)	Study 1 = 35 Psychology students	69%	They do not contribute	They do not contribute	They do not contribute
	Study2 = 473 College students	66.2%	They do not contribute	22.6	They do not contribute
**(3)** Bakir-Demir et al. (2021)	123 Psychology students	100%	18–25 years old	21.02	1.38
**(4)** Fido et al. (2020)	309	49.2%	18–66 years old	30.34	10.6
**(5)** Korpela et al. (2020)	Finland 301	86.7%	18–58	25.3	They do not contribute
	483	68.1%	17–86	38.9	They do not contribute
**(6)** Richardson and McEwan (2018)	*N* = 153	63.9%	18–75	45.78	11.74
**(7)** Sallay et al. (2023)	Finland: 259	87.6%	18–39	Finland= 23.93	4.36
	Hungary: 290	75.5%	18–40	Hungary= 28.96	6.17
**(8)** Theodorou et al. (2023)	187 students	80.2%		21.17	2.55
**(9)** Zhang et al. (2022)	977 general population	54.8%	21- 85 years old	They do not contribute	They do not contribute

Additionally, it is worth noting that the proportions of women were higher in most of the studies, except in three of them (1, 4, 9), where the sample was almost evenly distributed between men and women. In the study by Bakir-Demir et al. (2021), only the female sample was analyzed, despite initially having a larger sample, due to the low representation of men. This is likely because in most studies, the samples consisted of university students and in several cases (Table 2), psychology students (where the percentage of enrolled women is usually higher).

Secondly, only three studies used a sample from the general population. In the study by Fido et al. (2020), a power analysis was conducted beforehand to determine the appropriate sample size, and participants over the legal age were recruited through online surveys. In the study by Johnsen and Rydstedt (2013), visitors and hikers in rural areas were evaluated through on-site surveys. Richardson and McEwan (2018) conducted a complementary study using a larger sample from the general population to carry out a cross-sectional analysis of aspects related to the interest of the present review. It is also important to note that in the study samples by Korpela et al. (2020), the authors indicated that the Finnish sample consisted of students contacted via a mailing list, while in the Hungarian sample, participants were accessed through online platforms and personal networks of psychology students. The study by Sallay et al. (2023) was developed using a smaller selection from the same database.

### 3.3 Instruments and evaluated variables

As per the criterion for study selection, we collected the instruments used to measure nature contact and emotional regulation. Along these lines, we observed that only two studies used the same instrument to assess the construct of nature contact (3, 4), namely the Nature Relatedness Scale (NRS6; Nisbet et al., 2009). Additionally, Richardson and McEwan (2018) used an alternative measure to assess this same variable. The remaining studies used other indicators such as the provision of green spaces and exposure to these spaces (9), questionnaire to identify favorite places (5), physical characteristics of the place (7), or engagement with beauty (6).

Regarding the measurement of the emotional regulation variable, none of the studies coincide on the instruments used for evaluation. Some studies propose *ad hoc* measures, to assess emotional experience in specific places (1, 5, 7, 9). Among the validated questionnaires, we find the Difficulties in Emotion Regulation Scale (DERS-16; Bjureberg et al., 2016), the Emotion Regulation Questionnaire (ERQ; Gross and John, 2003), the Cognitive Emotion Regulation Questionnaire (CERQ, Garnefski and Kraaij, 2006), and positive and negative moods were measured using the Positive and Negative Affect Schedule (PANAS, Watson et al., 1988).

**Figure 2:**
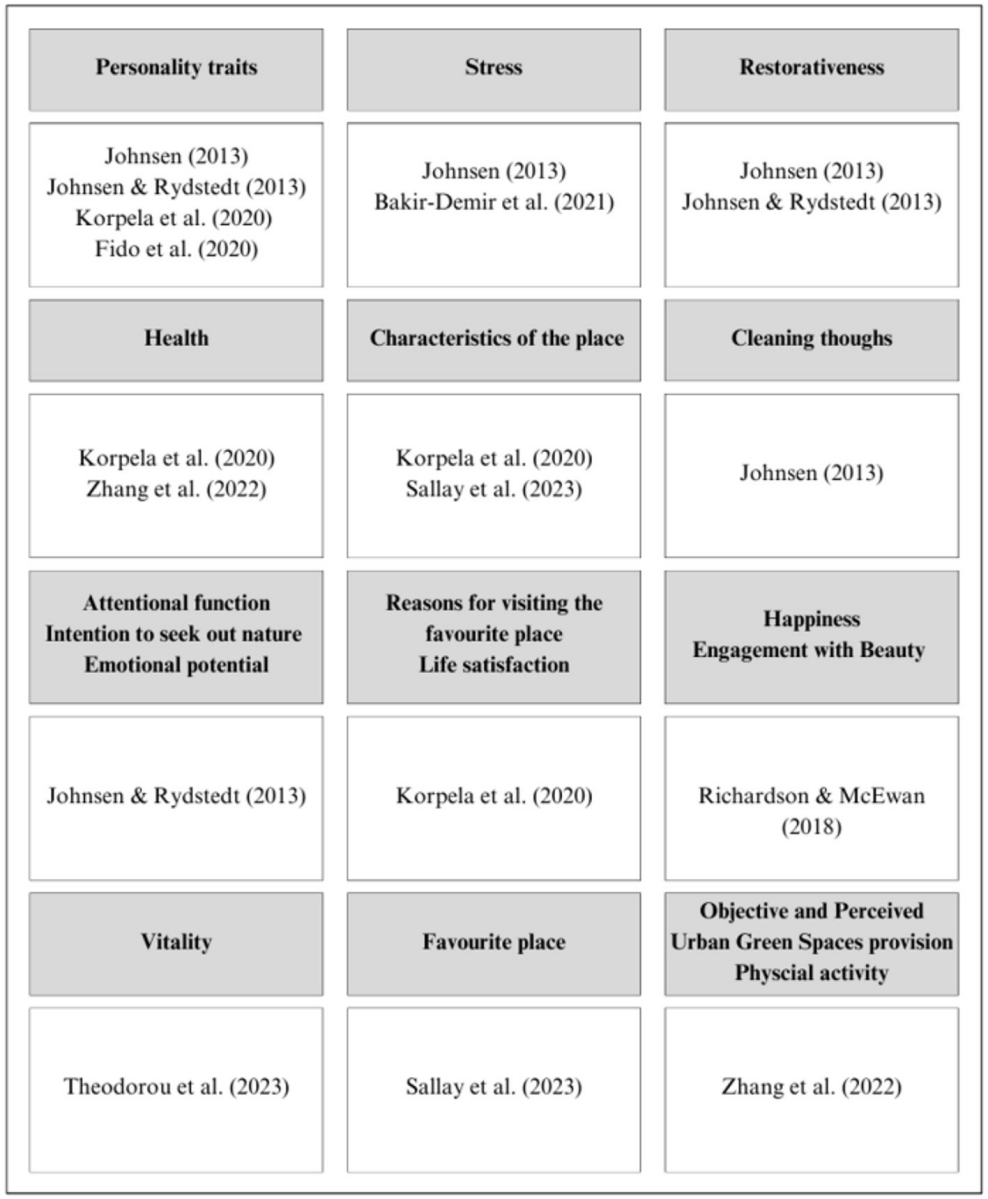
Other variables present in research into contact with nature and emotional regulation.

In addition to these fundamental variables, we have also considered other variables in research on nature contact and emotional regulation as summarized in Figure 2. Based on this synthesis, we conclude that personality variables such as neuroticism, conscientiousness, extraversion, Machiavellianism, narcissism, and psychopathy have aroused the greatest research interest. These are followed by stress, restoration capacity, health, and physical characteristics of environments. Some studies have also considered cognitive aspects such as mental clarity and attentional function, as well as motivational variables related to individuals' intentions to seek nature contact. Furthermore, variables related to psychological wellbeing, such as happiness, life satisfaction, and vitality, have also been studied.

### 3.4 Main findings

The main findings of the selected studies are presented in Table 3. Importantly, among the experimental studies, one of them used exposure to photographs of natural environments or other non-natural stimuli (Johnsen and Rydstedt, 2013), as well as virtual reality to explore different types of natural environments, such as parks, lakeside, arctic and urban environments (Theodorou et al., 2023). The other studies were cross-sectional, and only the study by Johnsen (2013) considered direct contact with nature. For more details, it is recommended to consult the Supplementary material.

**Table 3:** Characteristics and main findings of the nine studies included in the systematic review.

**Num. References**	**Design and aim**	**Variable(s)**	**Main results**
(1) Johnsen (2013)	Cross-sectional design. Analyzing how personality traits will influence the appraisal of nature, which in turn influences emotion regulation and affects restoration.	Personality ^a^ Perceived stress ^a^ Emotional regulation ^b^ Restoration ^c^	Negative emotion regulation was positively related to the evaluated restorative variables. Positive emotion regulation was also related to restorative effects (except for clearing of thoughts).
(2) Johnsen and Rydstedt (2013)	*Study 1*. Experimental. To test whether the natural environment increases positive mood and decreases negative mood. *Study 2*. Cross-sectional survey. To investigate the perception of different environments regarding emotional regulation and emotion-dependent motivational tendency to visit different environments.	*Study1:* Exposure to nature ^a^ Mood ^c^ Attentional Function ^c^ *Study 2:* Intention to seek out nature (emotional regulation) ^a^ Emotional potential of nature ^a^ *Personality (*Extraversion, Emotional Stability and Conscientiousness) ^e^ Gender ^e^ Mood Positive and Negative ^c^	*Study 1*. The use of nature to regulate emotions increases positive mood. *Study 2*. Classic nature scored significantly higher in emotional potential than the rest of the environments. Emotional potential correlated with the intention to seek nature when participants were happy.
(3) Bakir-Demir et al. (2021)	Cross-sectional design. Analyzing the mediating role of cognitive emotion regulation strategies in the relationship between connection with nature and stress.	Nature Connectedness ^a^ Cognitive emotion regulation (Adaptative and Non-adaptative) ^b^ Negative Reactivity ^e^ Hair cortisol ^c^ Stress Perceived ^c^	No direct effects of connection with nature were observed on perceived stress or accumulated cortisol. There is an indirect effect of connection with nature on perceived stress mediated by adaptive emotional regulation. Participants who are more connected with nature have better emotional regulation and lower levels of perceived stress.
(4) Fido et al. (2020)	Cross-sectional design. Investigating the moderating role of psychopathic personality in the relationship between connection with nature and emotional regulation.	Nature Connectedness ^a^ Psychopathy ^d^ Cognitive reappraisal ^c^ Expressive suppression ^c^	Connection with nature predicts the use of cognitive reappraisal strategies. Despite not being significant, there is an interaction between connection with nature and psychopathy.
(5) Korpela et al. (2020)	Cross-sectional design. Analyzing the links between motives/reasons for visiting favorite places, experiences in these places, and their connection with wellbeing, understood as the level of life satisfaction and perception of health.	Visit reasons favorite place ^a^ Positive recovery of Self ^b, c^ Low self-confidence and distress ^b, c^ Life Satisfaction ^a, c^ Perceived General Health ^a, c^ Place characteristics (natural vs. urban) ^e^	Visiting a favorite place in cases of reflective states or positive mood were stronger motives compared to experiencing sadness and depressive mood. Successful environmental self-regulation is not related to life satisfaction and perceived health.
(6) Richardson and McEwan (2018)	*Complementary study*: Cross-sectional design. Explore the relationship between changes in connection with nature, happiness, engagement with the beauty of nature, and emotion regulation.	Nature Connectedness ^a^ Engagement with Nature's Beauty ^b^ Emotion Regulation ^b^ Happiness ^c^ Health ^c^	Correlation analysis revealed that individuals experiencing difficulties in emotional regulation had a less pronounced connection with nature and experienced lower levels of happiness. Mediation analysis indicated that emotional regulation mediated the relationship between nature connectedness and happiness.
(7) Sallay et al. (2023)	Cross-sectional design. Examining the perceived physical characteristics of favorite places and the emotional experiences of those places.	Perceived physical characteristics of the favorite place ^a^ Gender and age ^e^ Favorite Places ^c^ Emotional Experiences in the favorite place ^c^	Preferences for favorite places involve perceptions and emotions of self-repair and distress. The results reveal that in both samples, individuals need favorite places to experience relatively high (non-clinical) distress. These are mixed places, including homes, nature, and urban destinations such as shops and communities.
(8) Theodorou et al. (2023)	Experimental design. Investigate the moderating role of the use of cognitive reappraisal strategy (as a mechanism of emotional regulation) in the relationship between exposure to virtual nature and subjective vitality.	Exposure to virtual natural environment ^a^ Sociodemographic ^e^ Personal conditions and individual differences (environmental identity, perceived stress) ^e^ Type of environment the participant lives ^e^ Variables impact the virtual reality experience ^e^ Pre-exposure subjective vitality ^e^ Cognitive reappraisal ^d^ Post-exposure subjective vitality ^c^	Presented natural environments (park, lakeside, and arctic) were significantly more effective than the urban environment in increasing levels of subjective vitality. Cognitive reappraisal can facilitate increases in subjective vitality from at least some types of nature exposure (lacustrine and arctic environment).
(9) Zhang et al. (2022)	Cross-sectional design. To determine whether exposure to UGS (Urban Green Spaces) is an independent variable or a mediator in the relationship between UGS and health	UGS provision ^a^ Perceived UGS ^a^ UGS Exposure ^a, b^ Green Physical Activities ^b^ Emotional regulation ^b^ Social interaction in UGS ^b^ General health ^c^ Mental health ^c^ Individual data ^e^	Three conceptual models addressing the relationships between Urban Green Spaces (UGS) availability or exposure and self-reported health were evaluated. Emotional regulation emerges as a mechanism to explain the mental health benefits of UGS

^a^independent variable(s); ^b^mediator variable(s); ^c^outcome variable(s); ^d^moderator variable(s); ^e^control/confounders variable(s).

Based on the findings of the studies, we can draw the following conclusions. First, Johnsen's (2013) shows that emotional regulation acts as a mediating factor between personality and restoration differently when it comes to positive or negative regulation. In other words, selecting situations (spending time in nature to experience positive emotions) versus modifying situations (spending time in nature to regulate negative emotions) affect different aspects of psychological restoration in each case. We conclude that the pursuit of nature contact is influenced by personality factors and by the motives for seeking that contact (increasing positive emotions or regulating negative emotions). Likewise, Johnsen and Rydstedt (2013) found that the use of nature increases positive mood and decreases negative mood, but in the latter case, no differences were found between natural environments and the other environments used in the research. Furthermore, they emphasize the idea that motives influence the search for environments for emotional regulation, a trend that is accentuated in the case of natural environments. They also highlight that there are gender differences in motives for seeking nature and in their effect on emotions. Similarly, Korpela et al. (2020) report that motives related to positive or reflective mood carry more weight than those related to sadness or depressive mood. Sallay et al. (2023) affirm that spaces where moderate distress can be experienced are in demand, as well as there being different preferences for selecting a type of environment according to other needs, such as restoration.

Second, regarding the effects of nature contact on aspects related to physical or mental health, Korpela et al. (2020) finds that environmental self-regulation is not related to life satisfaction or perceived health in their study. Nevertheless, Zhang et al. (2022) suggest that emotional regulation may explain the benefits of Urban Green Spaces for mental health. Similarly, Theodorou et al. (2023) found that cognitive reappraisal plays a moderating role between natural environmental types and subjective vitality. Furthermore, Bakir-Demir et al. (2021) conclude that a stronger connection with nature is associated with better emotion regulation and lower perceived stress. They also indicate that there is an indirect effect of nature contact on stress when emotional regulation strategies are adaptive. From the work of Fido et al. (2020), it can be inferred that connection with nature influences the use of cognitive reappraisal strategies, a type of emotional regulation that is more adaptive than others, such as expressive suppression. Additionally, Richardson and McEwan (2018) find that establishing a connection with nature is strongly associated with an adequate emotional regulation. In this study, the authors conclude that connection with nature is a key factor for wellbeing. This is the first evidence that establishes a relationship between affective regulation and the happiness benefits derived from such a connection.

### 3.5 Limitations identified by the authors in the examined articles

The authors note that in more than half of the studies, longitudinal research is considered necessary (Table 4). This is necessary to obtain information on how the relationship between nature contact and emotional regulation evolves over time. Likewise, it enables the detection of cause-and-effect relationships or patterns of stability or change in the variables.

**Table 4:** Limitations reported by the authors in the examined original articles.

This is a cross-sectional study, so causal relationships should be interpreted with caution. Need for longitudinal studies.	(Johnsen, 2013; Fido et al., 2020; Bakir-Demir et al., 2021; Sallay et al., 2023)
Results from a specific culture may not be generalizable.	(Johnsen, 2013; Korpela et al., 2020)
The sample consists of university students.	(Korpela et al., 2020; Bakir-Demir et al., 2021; Theodorou et al., 2023)
The sample is imbalanced in terms of gender (higher percentage of women).	(Richardson and McEwan, 2018; Korpela et al., 2020; Bakir-Demir et al., 2021; Theodorou et al., 2023)
Measurement of emotional regulation varied compared to recent literature.	(Fido et al., 2020)
Assessments are based on self-reported data.	(Fido et al., 2020; Zhang et al., 2022)
The sample size may be insufficient.	(Sallay et al., 2023)
Using only one type of emotional regulation strategy.	(Theodorou et al., 2023)

Other limitations are related to the composition of the sample. The authors identified at least three aspects to consider (see Table 4). First, it is noted that samples from a specific culture may not be generalizable, as they can lead to cultural and/or linguistic biases, lack of representativeness, cultural understanding all of which ultimately limiting the practical applicability of the results. Second, as the samples are predominantly composed of university students, which entails associated disadvantages such as demographic or selection bias, they present similar profiles of psychological, attitudinal, and behavioral characteristics and underrepresent other groups (older age groups or those with different educational levels). Third, an imbalance in the higher percentage of women compared to men in the samples is also mentioned. This poses the additional risk of interpreting findings based on characteristics of the predominant sex, generating potential gender bias. Finally, Sallay et al. (2023) indicates that the sample size may be insufficient.

Regarding the assessment, the authors point out two issues. First, the lack of consistency in the choice of assessment tools for emotional regulation can reduce validity when comparing results, make interpretation difficult, and limit the possibilities of comparison between studies. Second, Theodorou et al. (2023) emphasize the use of only one type of emotional regulation strategy, which similarly affects the comparison of results and their generalizability. Furthermore, the use of self-report measures is highlighted as a limitation by two of the articles analyzed (Fido et al., 2020; Zhang et al., 2022), along with the associated limitations in ensuring the validity and reliability of research results.

### 3.6 Brief summary of results

Firstly, upon examining the research questions posed in the selected studies, it has been noted that a significant number of these studies examine how the physical characteristics and emotional experiences in favorite places, as well as their active use, influence people's emotional regulation and wellbeing (Johnsen, 2013; Johnsen and Rydstedt, 2013; Korpela et al., 2020; Sallay et al., 2023).

Secondly, regarding the types of emotional regulation strategies addressed in the analyzed studies, cognitive reappraisal is the most studied emotional regulation strategy (Fido et al., 2020; Bakir-Demir et al., 2021; Theodorou et al., 2023), followed by situation selection and situation modification (Johnsen, 2013; Johnsen and Rydstedt, 2013), and positive self-recovery (Korpela et al., 2020; Sallay et al., 2023).

Thirdly, concerning the participants, the studies by Johnsen and Rydstedt (2013), Richardson and McEwan (2018), and Fido et al. (2020) have used a general population sample, which allows for obtaining generalizable results applicable to a wide range of individuals. Additionally, the diversity of the sample improves the robustness of the data and the practical applicability of the findings.

Fourthly, regarding the instruments used in these studies, it is observed that, on one hand, there is no unanimous criterion for measuring emotional regulation, although the use of the Emotion Regulation Questionnaire (ERQ) stands out (Fido et al., 2020; Theodorou et al., 2023). On the other hand, there is also no consensus on how to measure the connection with nature; however, Richardson and McEwan (2018) and Theodorou et al. (2023) use the Inclusion of Nature in Self (INS) scale.

Fifthly, the authors of the selected articles highlight the need for longitudinal research to better understand the relationship between contact with nature and emotional regulation (Johnsen, 2013; Fido et al., 2020; Bakir-Demir et al., 2021; Sallay et al., 2023). Additionally, certain limitations are noted, such as cultural biases, the predominance of university students, and a gender imbalance (Johnsen, 2013; Richardson and McEwan, 2018; Bakir-Demir et al., 2021; Theodorou et al., 2023).

Finally, regarding the main findings, it is worth noting: (1) the connection with nature is positively linked to the use of emotional regulation strategies such as cognitive reappraisal, reducing stress and improving individuals' wellbeing (Richardson and McEwan, 2018; Fido et al., 2020; Bakir-Demir et al., 2021); (2) exposure to natural environments, both real and virtual, enhances subjective vitality and positive mood, especially when using cognitive reappraisal as an emotional regulation strategy (Johnsen, 2013; Johnsen and Rydstedt, 2013; Theodorou et al., 2023); (3) the connection with nature and exposure to natural environments have mediating effects on mental and general health through stress reduction and improved emotional regulation (Johnsen, 2013; Bakir-Demir et al., 2021; Zhang et al., 2022); and (4) the physical characteristics and emotional experiences in favorite places influence emotional regulation and subjective wellbeing (Korpela et al., 2020; Sallay et al., 2023).

## 4 Discussion

This systematic review examines the relationship between contact with nature and emotional regulation, highlighting the following findings and their implications for environmental psychology. Specifically, literature analyzing both the impact that exposure to nature has on emotional regulation, and the role that emotional regulation plays in the relationship between psychological variables and contact with nature was reviewed.

The reviewed studies show some inconsistencies, which make it difficult to clarify the relationship between contact with nature and emotional regulation. Nevertheless, the significance of emotional regulation as a mediator between contact with nature and the resulting physical and mental health benefits is reinforced (Johnsen and Rydstedt, 2013). In this direction, Zhang et al. (2022) showed that emotional regulation is a key mediating mechanism between exposure to urban green spaces and mental health, although this result cannot be generalized to overall health. On the other hand, Theodorou et al. (2023) found that the strategy of cognitive reappraisal could act as a mediating variable between natural environments and subjective vitality. However, not all natural environments produce this effect to the same extent. Further exploration of the impact of environmental variables on wellbeing and mental health is needed.

The Stress Restoration Theory (Ulrich, 1993) proposes that exposure to nature decreases stress levels and promotes faster physiological and emotional recovery. Some of the analyzed studies (Johnsen, 2013; Bakir-Demir et al., 2021; Theodorou et al., 2023) include the stress variable. Specifically, the work of Bakir-Demir et al. (2021) did not show direct effects of connection to nature on perceived stress or accumulated cortisol. However, an indirect effect of connection to nature on perceived stress mediated by adaptive emotional regulation was found. In other words, those who are more connected to nature have better emotional regulation and lower levels of perceived stress.

The environmental self-regulation hypothesis by Korpela (1992) was addressed in three of the selected works. Specifically, the studies by Korpela et al. (2020) and Sallay et al. (2023) demonstrate that individuals are not only influenced by their environment but also choose environments that help them regulate their emotions. Similarly, Johnsen and Rydstedt (2013) indicate that the intention to seek nature is a strategy used by participants to modify their mood. These findings suggest that interaction with the environment not only influences emotions but also that the conscious choice of favorite places with specific characteristics (natural or urban) seems to be aimed at achieving emotional balance and subjective wellbeing. In other studies, included in this review, no evidence was found that individuals use the physical environment as an active tool to regulate their emotions by modifying their emotional state through interaction with the physical environment (Johnsen, 2013; Zhang et al., 2022).

Several studies suggest a correlation between contact with nature and improved emotional regulation (Richardson and McEwan, 2018; Fido et al., 2020). Specifically, Richardson and McEwan (2018) work, highlights the relevance of emotional regulation in connection with nature. The effect of nature could be influenced by the emotional strategy adopted and its effectiveness. This suggests that natural contact can yield varied results depending on the strategy used. However, not all natural environments have the same impact (Johnsen and Rydstedt, 2013). Effectiveness may vary depending on the type of natural environment and according to the underlying motives for seeking contact with nature. Exploring these differences allows us to identify which specific characteristics of natural environments are more beneficial for emotional regulation.

Similarly, in this direction, and based on the findings of Theodorou et al. (2023), it would be interesting to investigate to what extent exposure to a “real” vs. “virtual” natural environment generates changes in the way people emotionally self-regulate. In this sense, it is worth delving into the key components (being away, extent, fascination, and compatibility) for restoration, as postulated by the Attention Restoration Theory (ART) by Kaplan and Kaplan (1989) and Kaplan (1995), to identify which characteristics of natural environments provide the most benefits in terms of emotional regulation. Thus, Johnsen's (2013) work emphasizes that the regulation of self-reported positive and negative emotions in natural environments is related to the restorative benefits of such exposure.

In addition, this systematic review highlights the impact of certain personality traits in the way people experience and benefit from contact with nature (Johnsen, 2013; Johnsen and Rydstedt, 2013; Fido et al., 2020; Bakir-Demir et al., 2021). For example, individuals with dark personality traits, also known as the “dark triad,” which include Machiavellianism, psychopathy, and narcissism, may exhibit a different relationship between nature connection and any emotional regulation strategy that aims to emotionally reinterpret the meaning of an event or situation (Fido et al., 2020). These results suggest an in-depth analysis of the biophilia hypothesis (Kellert and Wilson, 1993), which suggests that the innate connection between humans and nature could be encoded in human genes. Therefore, it would be worth exploring the differences in connection with nature based on personality, as the existence of such differences could question or reformulate the hypothesis.

Most of the analyzed studies are based on the emotional regulation model by Gross and Thompson (2007). These authors proposed a theoretical model of emotional regulation that identifies five strategies deployed at different stages of an emotional experience: situation selection, situation modification, attentional deployment, cognitive change, and response modulation. Based on this proposal, it is possible to affirm that cognitive change, as indicated in the model, aligns with cognitive reappraisal, the most used emotional regulation strategy in the analyzed works (Fido et al., 2020; Bakir-Demir et al., 2021; Theodorou et al., 2023). Additionally, situation selection and situation modification are highlighted (Johnsen, 2013; Johnsen and Rydstedt, 2013), as well as positive self-recovery (Korpela et al., 2020; Sallay et al., 2023), which can be seen as a form of attentional deployment. Thus, the mentioned works emphasize the importance and practical application of the emotional regulation strategies described by Gross and Thompson (2007).

Furthermore, from this model (Gross and Thompson, 2007) it is considered emotional regulation as a dynamic process that involves the mutual influence of culture, context and individual strategies. In other words, this model attempts to provide an answer to how people regulate their emotions in intercultural contexts. In this way, it is necessary to highlight the cultural diversity present in the articles reviewed, as they cover a variety of countries with different socio-cultural contexts. This cultural diversity, in line with the theory of cultural dimensions (Hofstede, 2001), emphasizes the importance of considering cultural influences on individual perceptions and experiences with nature. Such diversity suggests that preferences and perceived benefits may vary considerably across cultures, underscoring the need for caution in generalizing results and designing nature-based interventions.

### 4.1 Limitations

This review also presents some limitations that must be considered when generalizing the conclusions. First, we must point out that the number of scientific publications directly addressing the relationship between contact with nature and emotional regulation is still limited. This lack of publications in turn limits the scope of the conclusions that can be drawn regarding whether contact with nature is beneficial for regulation processes and whether these processes mediate the impact of nature on people's wellbeing.

Second, although the criteria for reviewing only quantitative research have already been explained, the exclusion of qualitative research may have limited the evidence on the objectives of the review. Nevertheless, we consider that qualitative research would probably not yield conclusions different from those obtained here.

Finally, this review restricted eligibility to documents published in English and available in open access. This criterion may have excluded some relevant studies published in other languages and in other cultural contexts.

### 4.2 Future research

To enhance research on the relationship between contact with nature and emotional regulation, a primary need, almost an imperative, is to clarify the concept of emotional regulation. This requires a deeper exploration into the theoretical components of the concept. Likewise, a future line of work that would contribute to clarifying the relationship between emotional regulation and contact with nature would be to identify which specific characteristics of natural environments, such as size, quality, or accessibility, are most beneficial for emotional regulation. Similarly, the differential effects of population groups according to age, gender, and other social and cultural categories should be analyzed. In this same vein, it is necessary to increase the evidence on the benefits that contact with nature has for emotional regulation, using studies with a broader geographic, cultural, and socioeconomic representation of the populations and natural environments evaluated.

From the limitations identified in the studies reviewed, the lack of consistency in the choice of assessment tools, as well as the bias of the samples toward university populations and the high proportion of women pose important methodological challenges that should be addressed in future research. In addition, some areas for future research are suggested. The motives that drive people to seek out nature need to be explored, especially the emotions present when selecting a particular place. It seems that this factor is crucial both for assessing environment types and for determining the emotional regulation strategies adopted. For experimental study designs, we suggest considering measures or criteria related to the environmental quality of the environments, to investigate what objective characteristics might be influential. It would also be interesting for researchers to choose a longitudinal methodology, employ more representative samples and use appropriate assessment measures.

### 4.3 Conclusions

In conclusion, urban life can lead to a disconnection with nature due to its fast pace, high population density that reduces green spaces, lack of access to nature in some areas, prevalence of indoor entertainment, and decreased environmental awareness. This disconnection can negatively affect the health and wellbeing of both people and the environment. However, research on the relationship between contact with nature and emotional regulation provides valuable insights into understanding how natural environments can contribute to the emotional wellbeing and physical and mental health of the population. Thus, by addressing the identified limitations and exploring new research directions, it is possible to develop urban planning initiatives and mental health policies that promote the integration of green spaces in urban environments and the preservation of natural areas.

## Data availability statement

The original contributions presented in the study are included in the article/Supplementary material, further inquiries can be directed to the corresponding author.

## Author contributions

MR-R: Writing – review & editing, Writing – original draft, Visualization, Validation, Supervision, Software, Resources, Project administration, Methodology, Investigation, Funding acquisition, Formal analysis, Data curation, Conceptualization. CR: Writing – review & editing, Writing – original draft, Visualization, Validation, Supervision, Software, Resources, Project administration, Methodology, Investigation, Funding acquisition, Formal analysis, Data curation, Conceptualization. BH: Writing – review & editing, Writing – original draft, Visualization, Validation, Supervision, Software, Resources, Project administration, Methodology, Investigation, Funding acquisition, Formal analysis, Data curation, Conceptualization. ML: Writing – review & editing, Writing – original draft, Visualization, Validation, Supervision, Software, Resources, Project administration, Methodology, Investigation, Funding acquisition, Formal analysis, Data curation, Conceptualization.
