# Supplementary material for: Benefits for emotional regulation of contact with nature: a systematic review
Source: Front Psychol. 2024 Jul 19;15:1402885. doi: 10.3389/fpsyg.2024.1402885 (PMC11294936; doi:10.3389/fpsyg.2024.1402885)
Supplement: Supplementary file 1 [file Data_Sheet_1.docx]

Supplementary Material

Benefits for emotional regulation of contact with nature:

A systematic review

María Luisa Ríos-Rodríguez^1^, Christian Rosales^2*^, Bernardo Hernández^2^ and Maryurena Lorenzo^3^

*** Correspondence:** Corresponding Author: crosales@ull.edu.es

# Supplementary Figures and Tables

| **Table 1.** *Characteristics of included studies* | | | | | |
| --- | --- | --- | --- | --- | --- |
| **Nº studio**  **Authors/Year** | **Country** | **Aim & Design** |  | **Type of contact with nature** | **Outcome variable(s)** |
| (1) Johnsen (2013) | Norway | *Cross-sectional survey study.* Analyzing how personality traits will influence the appraisal of nature, which in turn influences emotion regulation and affects restoration. |  | Wild areas of Trollheimen and Jotunheimen. | - Personality (BFI-44). - Neuroticism, Conscientiousness, and Extraversion (Engvik & Føllesdal, 2005; Juan & Srivastava, 1999). - Perceived stress (Kamarck & Mermelstein, 1983). - Emotional regulation: - Regulation of negative emotions. - Regulation of positive emotions. - Approach motivation. - Nature appraisal. - Valuation. - Restoration. Restoration Outcome Scale (Korpela et al., 2008): - Relaxation. - Restoration of attention. - Clearing thoughts. - Restoration of ego. |
| (2) Johnsen & Rydstedt  (2013) | Norway | *Study 1* (Experimental): To test whether the natural environment increases positive mood and decreases negative mood.  *Study 2* (Cross-sectional survey): To investigate the perception of different environments regarding emotional regulation and emotion-dependent motivational tendency to visit different environments*.* |  | *Study 1:*  3 images  Experimental: Natural environment.  Control: Balloons.  Experimental 2: Natural environment, gentle instructions.  *Study 2:*  6 images  Urban environments with people, urban environments without people, "unsafe or frightening" natural environments, living rooms, shopping malls, and classic natural environments. | *Study 1:*   - Mood. Norwegian version of the Positive and Negative Affect Schedule (PANAS; Watson, Clark, & Tellegen, 1988). - Attentional function. Attentional Function Index (AFI; Cimprich, Visovatti, & Ronis, 2011). Comprising three factors: effective action (1), attention lapses (2), and interpersonal effectiveness (3).   *Study 2:*   - Extraversion, conscientiousness and neuroticism. Norwegian version of the Big Five Inventory (BFI-44; Engvik & Føllesdal, 2005; John & Srivastava, 1999). - Positive and negative moods. Positive and Negative Affect Schedule (PANAS) (Watson et al., 1988). - The intention to seek out nature was measured (ad hoc). - Emotional potential (four items, ad hoc). |
| (3) Bakir-Demir et al. (2021) | Turkey | Analyzing the mediating role of cognitive emotion regulation strategies in the relationship between connection with nature and stress (perceived and measures of accumulated cortisol). |  | Measures of connection with nature. | - Nature relatedness scale (NR) (Nisbet et al., 2009). - The cognitive emotion regulation questionnaire (CERQ) (Garnefski et al., 2001). - Negative Reactivity Subscale (Evans & Rothbart, 2007). - Perceived Stress Scale (PSS) (Cohen et al., 1983). - Hair cortisol. Concentration of cortisol in hair (HCC). - Stress Perceived. Adult Temperament Questionnaire (ATQ). |
| (4) Fido et al. (2020) | United Kingdom | Investigating the moderating role of psychopathic personality in the relationship between connection with nature and emotional regulation. |  | Self-reported measures of connection with nature. | - Nature Relatedness Scale (NRS6; Nisbet & Zelenski, 2013b). - Emotion Regulation Questionnaire (ERQ; Gross & John, 2003). - Short Dark Triad (SD3; Jones & Paulhus, 2014). |
| (5) Korpela et al. (2020) | Finland and Hungary | Analyzing the links between motives/reasons for visiting favorite places, experiences in these places, and their connection with well-being, understood as the level of life satisfaction and perception of health. |  | Favorite urban and/or natural places. | - Place characteristics (natural vs. urban). - Visit motives (ad hoc). - Experiences (ad hoc). Positive recovery and low self-confidence/distress. - Satisfaction With Life Scale (SWLS; Diener, Emmons, Larsen, & Griffin, 1985); for the Hungarian version (Martos, Sallay, Désfalvi, Szabó & Ittzé, 2014). - Perceived general health was measured by a widely-used single question (Bronzaft, Ahern, McGinn, O'Connor, & Savino, 1998). |
| (6) Richardson & McEwan (2018) | United Kingdom | In a larger research endeavor, a complementary study is conducted to explore the relationship between changes in connection with nature, happiness, engagement with the beauty of nature, and emotion regulation. |  | Measures of connection with nature and engagement with beauty. | - Difficulties in Emotion Regulation Scale: DERS-16 (Bjureberg et al., 2016). - Engagement with Beauty scale developed (EWBN) (Diessner et al., 2008). - Single-item measures for happiness and connection with nature (Schultz, 2001). |
| (7) Sallay et al. (2023) | Finland and Hungary | Examining the perceived physical characteristics of favorite places and the emotional experiences of those places. An approach is adopted to identify patterns that intersect both aspects (the type of place and emotional experience), as well as to compare samples from two different countries. |  | Selection of favorite places of different typologies. | - The Favorite Places Questionnaire (Korpela et al., 2020; Korpela & Ylén, 2007). - Perceived physical characteristics of the favorite place (ad hoc). - Experiences in the favorite place (ad hoc). |
| (8) Theodorou et al. (2023) | Italy | Investigate the moderating role of the use of cognitive reappraisal strategy (as a mechanism of emotional regulation) in the relationship between exposure to virtual nature and subjective vitality. Panoramic photographs in four environments (virtual reality): |  | National park  Lakeside environment  Arctic environment  Urban environment | *Main variables*:   - Cognitive reappraisal: Emotion Regulation Questionnaire (Balzarotti, 2021, original instrument Gross & John, 2003). - Subjective vitality: The State Subjective Vitality Scale (Ryan & Frederick, 1997; Bostic et al., 2021).   *Control variables*:   - Sociodemographic variables. - Environmental identity: Nature in Self (INS) (Schultz, 2002). - Perceived Stress Scale (PSS) (Cohen et al., 1983). - Type of environment where the participant lives (one question). - Previous experience in virtual reality (one question). - Brightness of the images. - Sense of presence: I group Presence Questionnaire (IPQ) (Schubert et al., 2001). - Motion sickness: Motion Sickness Assessment Questionnaire (MSAQ) (Gianaros et al., 2001). |
| (9) Zhang et al. (2022) | Singapore | To determine whether exposure to UGS is an independent variable or a mediator in the relationship between UGS and health |  | Self-reported measures about Urban Green Spaces (UGS) are used | - Self-reported health: General Health Questionnaire (GHQ-12) (Tsurumi et al., 2018; Dadvand et al., 2016; Lim et al., 2005; Satghare et al., 2016) and self-reported general health measured by a single General Self-Rated Health (GSRH) question (DeSalvo et al., 2006). - Objective provision of Urban Green Spaces (UGS): Calculation of the quantity of parks, vegetation, and canopy coverage. - Subjective provision of UGS: Perception of quantity, accessibility, and quality of use. - UGS exposure: Time spent visiting green spaces and frequency of use. - Physical Activity: Godin-Shephard Leisure-Time Physical Activity Questionnaire (Amireault et al., 2015). - Emotional regulation: Satisfaction and relaxation items. - Social interaction in UGS. - Demographic, socioeconomic, and other individual data. |

| **Table 2.** *Analyzed data and main results* | | |  |
| --- | --- | --- | --- |
| **Nº studio** | **Data analysis** | **Results** | |
| (1) | Pearson correlation analysis of key variables.  Exploratory factor analysis, confirmatory  factor analysis for measurement scales.  Structural equation models. | Negative emotion regulation was positively related to the evaluated restorative variables (relaxation, clearing of thoughts, restoration of attention, and restoration of ego).  Positive emotion regulation was also related to restorative effects (except for clearing of thoughts).  A 44% variance of restoration is explained by a dual pathway: 1) Negative emotional regulation as a mediator between neuroticism and restoration; 2) Positive emotional regulation and nature appraisal as mediators between extraversion and conscientiousness with restorative effects. | |
| (2) | *Studio 1:*  Repeated measures ANOVA.  *Studio 2:*  Paired samples t-test and regression | **Study 1**  The use of nature to regulate emotions increases positive mood. Although a decrease in negative mood was observed, there were no differences between the groups.  There are indications that nature has effects on cognitive functioning (restoration of ego).  **Study 2**  Classic nature scored significantly higher in emotional potential than the rest of the environments.  Emotional potential correlated with the intention to seek nature when participants were happy. Likewise, a strong motivational tendency to seek environments to regulate oneself when participants were sad was observed, and this tendency was more pronounced in the case of classic natural environments.  Gender differences were observed. For men, the intention to seek nature when they were happy was a significant predictor of positive mood, while for women, the intention to seek nature when they were sad predicted positive mood. | |
| (3) | Correlations between variables.  Mediation analysis. | No direct effects of connection with nature were observed on perceived stress or accumulated cortisol.  There is an indirect effect of connection with nature on perceived stress mediated by adaptive emotional regulation (this is not replicated when the mediating variable is maladaptive emotional regulation).  Participants who are more connected with nature have better emotional regulation and lower levels of perceived stress. | |
| (4) | Pearson correlations.  Moderation models. | Connection with nature predicts the use of cognitive reappraisal strategies.  Despite not being significant, there is an interaction between connection with nature and psychopathy, such that high levels of psychopathy imply less association between connection with nature and cognitive reappraisal.  In contrast, neither connection with nature nor the interaction explained the expressive suppression strategy. | |
| (5) | Correlation analysis.  Exploratory Factor Analysis (EFA).  Structural Equation Modeling (SEM). | The majority (56%) of favorite places in this sample of adults were natural environments.  Visiting a favorite place in cases of reflective states or positive mood were stronger motives compared to experiencing sadness and depressive mood.  Successful environmental self-regulation is not related to life satisfaction and perceived health.  Conversely, it was found that life satisfaction is significantly associated with two types of reasons for going to a favorite place ("sad, depressed" and "happy, well"), but no relationship was found with perceived health. | |
| (6) | Pearson correlations.  Mediation analysis. | Correlation analysis revealed that individuals experiencing difficulties in emotional regulation had a less pronounced connection with nature and experienced lower levels of happiness.  Mediation analysis indicated that emotional regulation mediated the relationship between nature connectedness and happiness. | |
| (7) | Exploratory Factor Analysis, Confirmatory Factor Analysis, Latent Profile Analysis (LPA) for pattern-oriented analysis. Multinomial logistic regression analysis. | Preferences for favorite places involve perceptions and emotions of self-repair and distress.  In latent profile analysis of these experiences, subgroups were identified: in the Finnish sample, the largest groups show preferences for "homely environment for distress and recovery" and "self-restoration in nature." In the Hungarian sample, the most prominent subgroups prefer "natural environment for external attention" and "urban/built environment for restoration."  The results reveal that in both samples, individuals need favorite places to experience relatively high (non-clinical) distress. These are mixed places, including homes, nature, and urban destinations such as shops and communities. | |
| (8) | One-way ANOVA with chi-square tests.  Multicategorical mediation analysis. | Presented natural environments (park, lakeside, and arctic) were significantly more effective than the urban environment in increasing levels of subjective vitality.  Cognitive reappraisal had no direct effect on vitality but depends on interaction with the environment. In this line, it was observed that:   - Cognitive reappraisal only had a mediating effect in lakeside and arctic environments. - In these environments, individuals showing low levels of cognitive reappraisal did not obtain significant effects after exposure to virtual nature (compared to urban environments). Conversely, in participants with high levels of cognitive reappraisal, these effects were found. | |
| (9) | Path analysis and Structural Equation Models (SEM). | Three conceptual models addressing the relationships between Urban Green Space (UGS) availability or exposure and self-reported health were evaluated.  Emotional regulation emerges as a mechanism to explain the mental health benefits of UGS | |
